# Toward Realistic Modeling of Complex Semiconductors

**DOI:** 10.1021/acsmaterialsau.6c00150

**Published:** 2026-09-09

**Authors:** Julia Wiktor

**Affiliations:** 11248 Chalmers University of Technology, Department of Physics and Astronomy, Gothenburg 41296, Sweden

**Keywords:** atomistic modeling, structural disorder, finite-temperature effects, charge localization, defects, interfaces, machine-learned interatomic potentials

## Abstract

Complex semiconductors are increasingly used in energy conversion, optoelectronics, and photocatalysis. Their performance is often controlled by local atomic environments that are not represented by a single ideal crystal structure. In this Perspective, I discuss how atomistic models can include effects such as thermal motion, disorder, charge localization, defects, and interfaces when these are relevant to the property of interest. I focus on examples where including these effects changes the physical interpretation of the calculation, and discuss how machine-learned interatomic potentials can help sample the relevant configurations for subsequent electronic structure calculations.

## Graphical abstract

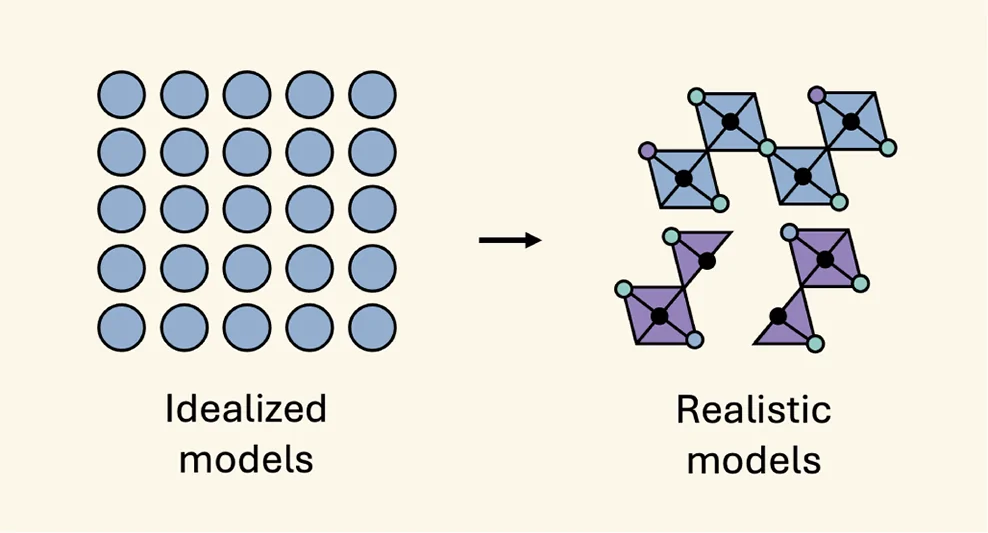

## 1 Introduction

The development of new semiconductors for energy and optoelectronic applications requires understanding how microscopic structure controls macroscopic performance. This connection is particularly important when functionality is affected in non-trivial ways by complex atomic-level phenomena. For example, in the case of metal halide perovskites, soft lattices and compositional flexibility are among the reasons for their success in photovoltaics, light emission, and radiation detection.^1^ The same features, however, also make them susceptible to phase instabilities, ion migration, compositional segregation, and degradation. Complex metal oxides are another example, where multiple oxidation states, defects, or surface effects can determine photo- and electrocatalytic behavior.^2,3^ Chalcogenides, oxyhalides, molecular semiconductors, and metal–organic frameworks are further cases of structurally complex materials with promising applications,^4–8^ where we are still working to establish the connections between the atomic and device scales.

Atomistic calculations can identify mechanisms that are difficult to isolate experimentally. They can show how a particular change in structure modifies a band edge, how a defect introduces a trap state, how a surface binds a reaction intermediate, or how an excess charge couples to the lattice. However, conclusions can only be drawn about the effects that are explicitly included in the model. A single relaxed crystal structure is often a useful reference, but it may not represent the local environments present in real materials, where finite-temperature motion, compositional disorder, defects, interfaces, or charge localization play a role. The appropriate level of realism depends on the property of interest, as the model should include the effects most likely to change the conclusion. Examples of such effects are summarized schematically in Figure 1.

**Figure 1:**
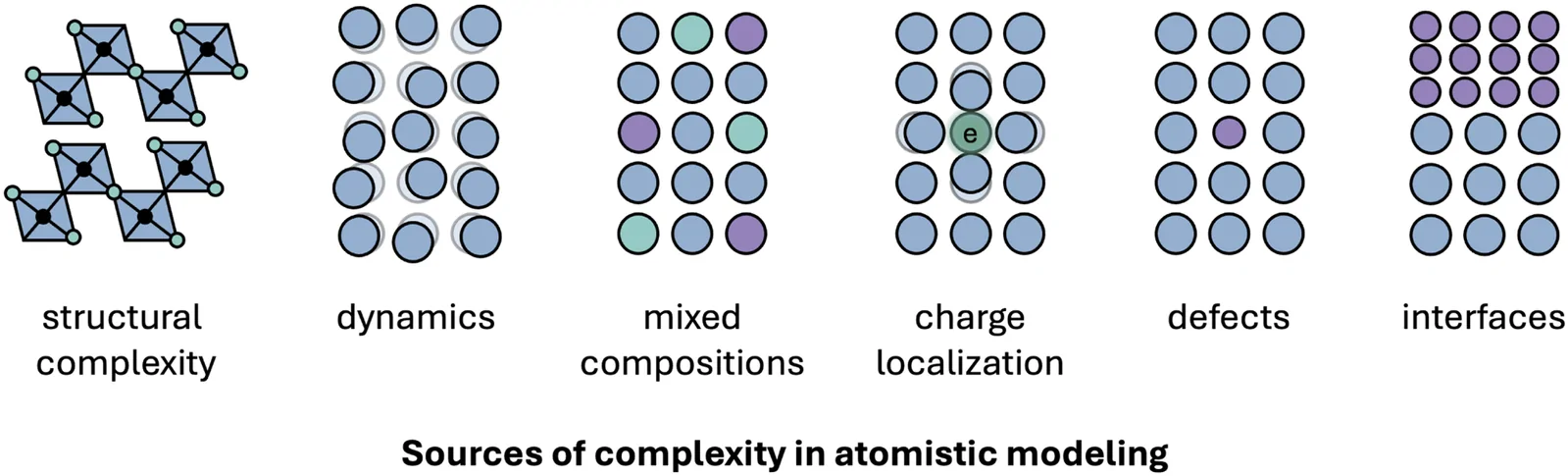
Schematic illustration of effects that can be included in realistic models of complex semiconductors, including structural complexity, finite-temperature dynamics, compositional disorder, charge localization, defects, and interfaces.

In this Perspective, I discuss several cases where these effects have to be included explicitly to obtain a meaningful atomistic description. I focus on recurring modeling problems in complex semiconductors: how to define realistic structures, how to include finite-temperature dynamics and mixed compositions, how to describe localized charges and defects, and how to construct useful interface models. The examples are selective and focus primarily on halide perovskites and complex oxides, reflecting areas in which I have worked most extensively. I also discuss the role of machine-learned interatomic potentials, which have become increasingly important for sampling the structural configurations needed in such calculations.

## 2 Structural Complexity

The first practical question in an atomistic calculation is which structure to use. For simple crystalline materials, this choice is often straightforward: a well-resolved experimental structure can be used directly, potentially after an initial relaxation. In complex materials, the structural model can itself become one of the main sources of uncertainty or computational cost. Sometimes, the structure is known, but the unit cell is already large due to a complex chemical formula or low symmetry. In other cases, the average crystallographic structure is known but is too simple to describe the local environments relevant to the property of interest. Finally, the experimentally realized phase may itself be uncertain, metastable, or dependent on synthesis and thermal history. I refer to these cases collectively as structural complexity, separately from the compositional disorder and point defects discussed below.

When the average structure is known but local atomic displacements from ideal crystallographic positions are important, larger structural models are often needed. Local symmetry breaking can strongly affect fundamental properties. Recently, increasing attention has been given to treating such cases using so-called *polymorphous* structures.^9^ This approach has been applied to halide perovskites, paraelectric oxides, and paramagnetic transition-metal oxides, where enforcing the highest-symmetry primitive cell can give qualitatively wrong electronic structures.^9–11^ Figure 2 schematically compares an average crystallographic structure, a static polymorphous structure, and a finite-temperature structural ensemble, and illustrates how these representations can lead to different descriptions of a structure-sensitive property such as the band gap.

**Figure 2:**
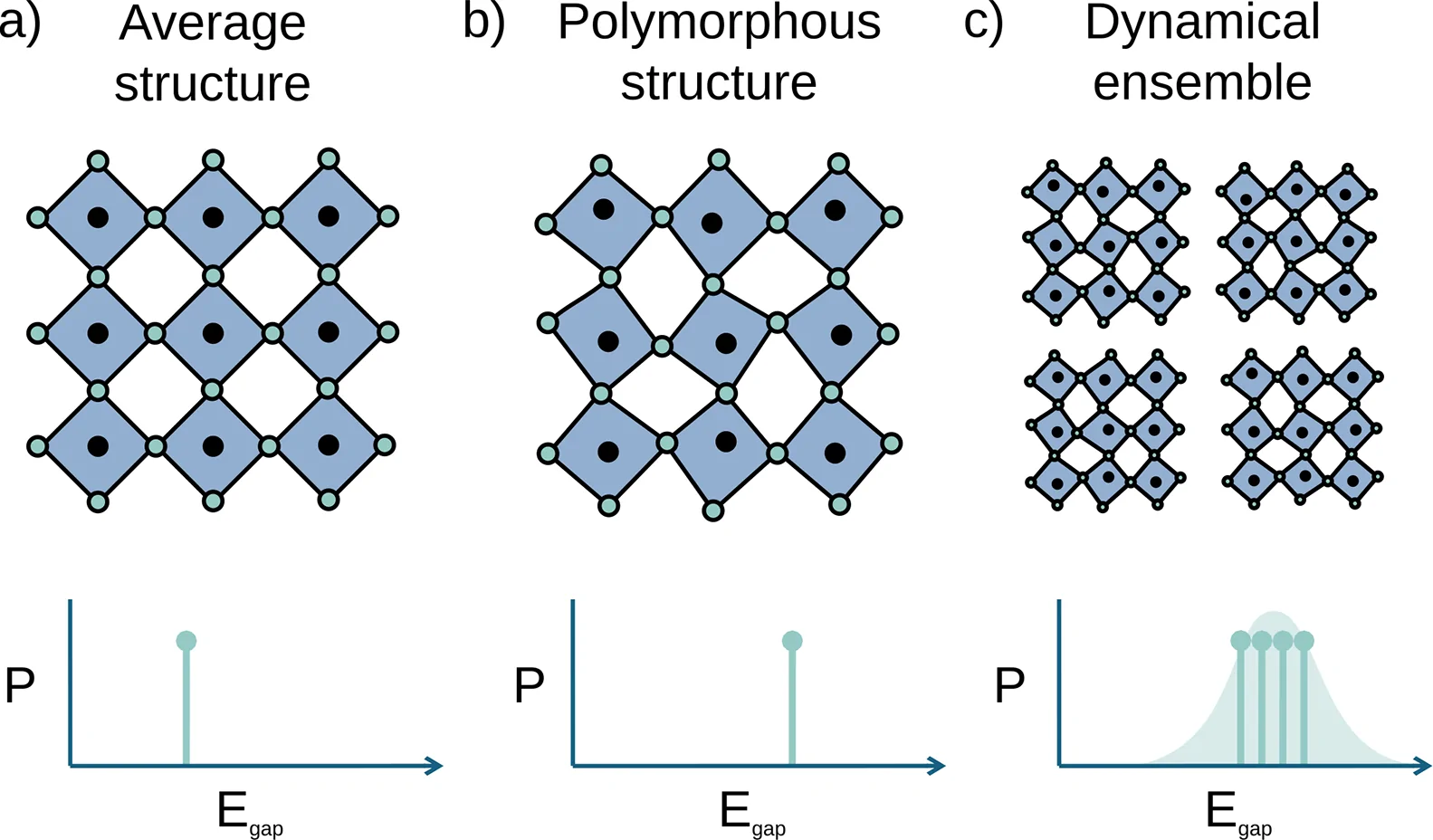
Schematic effect of structural representation on the calculated band gap: (a) average high-symmetry structure, (b) polymorphous structure, and (c) finite-temperature ensemble. Vertical lines indicate values for individual structures, while the shaded area represents the band-gap distribution. *P* denotes probability density.

A different problem occurs when the relevant phase is not known in advance. One example is the family of silver–bismuth iodides, where structures inferred from nominal composition and diffraction have required reassessment using first-principles calculations.^12^ In such cases, finding a suitable structural model can become the main modeling problem rather than a preliminary step. Sometimes this can be solved by comparing a small number of chemically plausible structures against measured properties such as diffraction patterns. When the configurational space is larger, global structure-search methods, including evolutionary algorithms, particle-swarm optimization, and random structure searching, provide more systematic ways of identifying candidate stable and metastable structures.^13–15^

Hybrid organic–inorganic materials, such as metal halide perovskites, can combine several of the aforementioned difficulties. They contain molecular cations with rotational degrees of freedom, which often correspond to relatively flat potential-energy surfaces and are coupled to phase transitions. As a result, determining the ground-state structure for calculations at 0 K can be difficult, and the ground state itself may be a poor representation of the finite-temperature material. Formamidinium lead iodide, FAPbI_3_, is a good example of this issue. Despite its importance for high-efficiency perovskite solar cells, its low-temperature structure has remained difficult to resolve.^16^ In our recent work, a machine-learned potential trained on DFT was used to search a large configurational space and perform finite-temperature molecular dynamics (MD). Using Glazer notation for the octahedral tilts,^17^ that study identified an *a* ^–^ *b* ^–^ *b* ^–^ ground state but found that FAPbI_3_ becomes kinetically trapped in a metastable *a* ^–^ *a* ^–^ *c* ^+^ phase upon cooling. The trapping was attributed to large barriers associated with both reorienting the formamidinium cations and transforming the octahedral tilt pattern required to reach the thermodynamic ground state.^18^ A subsequent study proposed a kinetically arrested twin-domain state in FAPbI_3_, further illustrating that even advanced simulations and experiments may not immediately reduce complex materials to a single unambiguous structural model.^19^

## 3 Dynamical Effects

Finite-temperature effects become especially important when a material has a soft lattice, where low-energy distortions allow relatively large atomic displacements. The potential-energy surface may also be strongly anharmonic, meaning that it deviates substantially from a quadratic form. When thermal motion produces large time-dependent fluctuations of the local atomic structure, this is commonly described as dynamical disorder. Thermally sampled structures can affect calculated band gaps, absorption spectra, and other properties. For example, in BiVO_4_, thermal vibrations and nuclear quantum motion contribute substantially to the calculated optical response.^20^ Metal halide perovskites are a prominent example of strong dynamical disorder associated with soft, anharmonic lattice dynamics.^21^ Their band gaps are sensitive to the instantaneous atomic structure, and calculations based only on idealized 0 K geometries can be a poor representation of their properties at operating temperatures.^22,23^ It has been shown that anharmonic thermal fluctuations and electron–phonon coupling are essential for describing the temperature dependence of the band gap and electronic structure in these materials.^24,25^

The influence of dynamics is not limited to band energies. In halide perovskites, coupling to soft polar modes affects carrier mobility, carrier cooling, and recombination. Photoluminescence measurements and first-principles calculations have shown that longitudinal optical phonons dominate room-temperature electron–phonon coupling,^26^ while large-polaron models describe mobility as limited by polar-optical phonon scattering.^27^ Dynamic disorder has also been shown to influence carrier localization, transport, and recombination.^28^

The importance of anharmonic and dynamical effects extends well beyond halide perovskites. In the nitride semiconductor CuTaN_2_, anharmonic lattice dynamics strongly affect the band gap and thermal expansion.^29^ In Sb_2_S_3_, first-principles calculations show strong electron–phonon coupling and a temperature-induced renormalization of the absorption edge by about 0.2 eV between 10 and 300 K.^30^ Small-molecule organic semiconductors such as anthracene and pentacene show temperature-dependent symmetry breaking of lattice vibrations,^31^ while anharmonic exciton–phonon coupling has been reported in metal–organic chalcogenide quantum wells.^32^ In the oxide perovskite SrTiO_3_, explicit anharmonic sampling substantially modifies the finite-temperature band gap and effective masses.^33^

The appropriate computational treatment of dynamical effects depends on the character of the nuclear motion and on the target property. Perturbative phonon and electron–phonon approaches are efficient when a harmonic reference remains meaningful. Special-displacement methods can provide efficient finite-temperature configurational averages that include zero-point motion and quantum vibrational statistics through Bose–Einstein phonon occupations, and can be combined with self-consistent treatments of anharmonicity.^34,35^ Molecular dynamics can explicitly sample nuclear trajectories and capture strongly anharmonic fluctuations, but requires convergence with respect to supercell size and sampling time. Although classical molecular dynamics omits nuclear quantum effects, these can be included in equilibrium configurational sampling using path-integral molecular dynamics, which additionally requires convergence with respect to the number of path-integral beads.^36^ Temperature-dependent electronic properties can then be obtained by averaging over representative configurations, although this can become computationally expensive when each configuration requires a high-level electronic-structure calculation. For optical properties, several levels of theory may have to be combined. In BiVO_4_, for example, quantitative modeling of the absorption spectrum required *GW* quasiparticle corrections, Bethe–Salpeter calculations of electron–hole interactions, and nuclear-motion effects to be considered together.^20^

Many complex semiconductors undergo structural phase transitions within relevant temperature ranges, making it necessary to consider not only a single low-temperature structure but also how the material evolves with temperature. Machine-learned interatomic potentials have recently made it possible to directly study such transitions in large supercells and over long simulation times. These simulations can be used to map phase diagrams and investigate how predicted transition temperatures depend on factors such as system size, heating rate, model uncertainty, and the underlying exchange–correlation functional.^37,38^

Beyond identifying transition temperatures, large-scale simulations provide information about the microscopic nature of the phases themselves. In halide perovskites, phases that are classified crystallographically by their average symmetry can still exhibit substantial local distortions and correlated atomic motion. Experimental and computational studies of CsPbBr_3_ have shown that local structural fluctuations remain correlated over several unit cells, even in phases that appear highly symmetric on average,^39^ while molecular dynamics simulations reveal correlated octahedral tilting even in phases that are cubic on average.^38,40^ Such behavior illustrates that even an experimentally determined average crystallographic structure may provide an incomplete picture of the local environments sampled at finite temperature.

The same finite-temperature structural ensemble can also affect quantities that are usually introduced separately, including charge localization and defect thermodynamics. These are discussed in the following sections.

## 4 Mixed Compositions

Many complex semiconductors are used as mixed materials rather than as single, ordered compounds. In halide perovskite solar cells, the best-performing absorbers often contain mixtures of A-site cations and halides.^41^ Mixing is used as a strategy to, for example, enhance stability or tune the band gap. From the computational point of view, this substantially increases the complexity of the modeling problem. The difficulty is that the chemical formula gives only the average composition. An atomistic calculation, however, requires choosing a particular arrangement of atoms in a finite cell. A single ordered unit cell may be useful as a starting point, but it does not explicitly represent the distribution of local environments present in a random or partially ordered alloy. Larger cells are often needed to include a representative distribution of local environments. This becomes even more important when mixing is combined with other effects, such as localized charges^42^ or defects,^43,44^ which depend on the local chemical environment.

Several approaches can be used to treat mixed compounds in atomistic simulations. Special quasirandom structures (SQS) can approximate a disordered alloy in a finite supercell,^45^ while cluster expansions can be used to study configurational energetics, ordering, and phase diagrams.^46,47^ Machine-learned interatomic potentials can also be used together with molecular dynamics and Monte Carlo methods to sample mixed compositions in large cells and at finite temperatures. These approaches are useful not only for generating structures, but also for deciding what kind of structural model is appropriate in the first place: a random alloy, an ordered phase, or a mixture of competing phases.

This type of phase exploration has recently been applied to mixed cation and mixed halide perovskites. In FA_1–*x*_MA_*x*_PbI_3_, simulations based on a neuroevolution potential^48^ were used to map out the phase diagram and revealed the emergence of morphotropic phase boundaries.^49^ In another study, large-scale molecular dynamics based on a machine-learned force field was used to study CsPb*X*_3_, MAPb*X*_3_, and FAPb*X*_3_ across temperatures and halide mixtures. The simulations showed that the A-site cation strongly modulates octahedral tilt modes and phase-transition pathways, while changes in halide composition and arrangement, including partial segregation, alter tilt correlations and structural transformations.^50^

One methodological aspect of modeling configurational disorder is convergence with respect to cell size and sampling. The required cell size and amount of sampling depend on the property of interest. An SQS that reproduces short-range pair correlations well may still be insufficient for properties that depend on particular local environments or longer-range correlations.^45^ For finite-temperature Monte Carlo or molecular dynamics, the cell and sampling time also have to be sufficient to explore the relevant rearrangements.^37,47^

The importance of mixing is not limited to halide perovskites. In oxide perovskites, it appears in a particularly clear form at morphotropic phase boundaries, where large responses arise from the competition between nearly degenerate local structural motifs.^51,52^ Further, in high-entropy semiconductors, oxides, and hybrid perovskites, disorder is part of the design strategy: mixing several species on the same sublattice can be used to tune stability and properties.^53,54^ Mixed-anion oxynitrides are another example, because O and N share the anion sublattice and their local arrangement can affect the electronic structure and surface properties.^55^

## 5 Localized Charges

Excess or photoexcited carriers in semiconductors are often assumed to occupy delocalized states within or close to the band edges. In soft and polarizable materials, this may not be the lowest-energy configuration. Electrons and holes can couple to lattice distortions to form polarons, while bound electron–hole pairs can form self-trapped excitons. These localized states affect fundamental properties of the material in multiple ways, for example by modifying the charge mobility, recombination, emission, band alignment, and photocatalytic reactivity.^56^ Their description can also depend on practical modeling choices, including the initial distortion, supercell size, exchange–correlation functional, and treatment of electrostatic finite-size effects.

Modeling localized charges requires particular care in the choice of the electronic-structure method. Semilocal density functionals generally suffer from self-interaction error, which can artificially favor delocalized solutions. Hybrid functionals, DFT+*U*, Koopmans-based approaches, or local-potential corrections are therefore often used to address this problem.^56–62^

For polarons, first-principles calculations can be divided broadly into two approaches. In direct supercell calculations, the localized carrier and the accompanying lattice distortion are represented explicitly. This approach can be directly used when localization occurs at defects, surfaces, or interfaces, but requires convergence with respect to supercell size and, for charged cells, treatment of electrostatic finite-size effects. An alternative is to formulate polaron formation in terms of the electron–phonon coupling of the periodic crystal. Several approaches have been developed to obtain polaron energies and wave functions without constructing an explicit large distorted supercell. These include energy-functional, canonical-transformation, variational, and many-body formulations.^63–66^ These approaches can describe both localized and more extended polarons, but require careful convergence of the electronic and phonon wave-vector sampling and are most straightforward for periodic crystals. Related formulations based on first-principles exciton–phonon coupling have also been developed for self-trapped excitons.^67^

Even when a localized state is identified, connecting it to a measurable property usually requires an additional calculation. In photocatalysis, for example, reaction steps have to be calculated in the presence of the localized charge.^68,69^ For transport, hopping barriers can be estimated with methods such as nudged elastic band calculations,^70^ but this gives an approximate picture whose validity depends on the degree of adiabaticity and temperature dependence of the barriers. Direct finite-temperature simulations of polaron motion are desirable, and machine-learned models have recently been used to explore small-polaron hopping in MgO and TiO_2_.^71^ Nevertheless, a generally applicable treatment of explicit excess charges remains difficult.

In complex materials, the question is often not only whether a charge localizes, but how many localization modes are available and which of them are relevant under operating conditions. When the local symmetry is low, the same excess charge can localize on different sites or with different spatial distributions. If two charges are involved, as in double polarons or self-trapped excitons, there are additional possibilities because the relative arrangement of the two localized charges also matters. For example, in tin halide perovskites, excess electrons can form strongly stabilized localized states, including double-polaron configurations, which helps explain why charge localization can be detrimental for lead-free absorber performance.^72,73^ Germanium halide perovskites, CsGeCl_3_, CsGeBr_3_, and CsGeI_3_, show an even larger configurational space due to the lone-pair activity and off-centering of Ge, with several possible polaron and self-trapped exciton configurations.^74^

A similar situation appears in photocatalytic materials with low-symmetry or layered structures. In BiVO_4_, self-trapped excitons can occupy different local environments, and competing self-trapped exciton configurations lead to multiple emission pathways.^75,76^ In bismuth oxyhalides, BiO*X*, the hole polaron can localize in different parts of the layered structure depending on the halide chemistry.^77^ In Bi_4_TaO_8_ *X* oxyhalides, the layered structure similarly gives rise to several possible hole-polaron configurations.^78^

## 6 Defects

Point defects are unavoidable in real materials and may therefore need to be considered in realistic atomistic models. They can trap carriers and introduce recombination pathways,^79,80^ mediate ion migration,^81^ determine carrier concentrations,^82^ or modify surface reactions.^69^ Defect calculations have therefore become a standard part of computational semiconductor research, and the field has converged on a set of well-established methodologies, with reviews and best-practice papers providing guidance for accurate calculations.^79,80,83^ Standard workflows require convergence with supercell size, treatment of electrostatic finite-size effects for charged defects, appropriate chemical potentials, and an electronic-structure method that describes the relevant band edges and localized states.^80,83^ For quantities involving defect levels and localized lattice relaxations, the finite-size correction itself can require additional care because ionic polarization contributes to the finite-size error in addition to electronic screening.^84^ Here I focus on cases where realistic structural modeling changes either the assigned defect structure or the physical role of the defect.

Structural ambiguity can also arise in defect calculations. In complex semiconductors, a defect may have several low-energy geometries, so its calculated properties can depend on which structure is considered.^85^ Structure-search workflows can help identify low-energy defect configurations that may be missed by standard relaxations.^86^ More generally, defect stability should be formulated in terms of free energies, including vibrational, configurational, electronic, and spin contributions.^87^ Including finite-temperature effects is becoming more practical: machine-learned interatomic potentials trained for different defect charge states can now be used to compute finite-temperature charge transition levels, as recently shown for vacancies in MgO, LiF, and CsSnBr_3_.^88^ These calculations show that a defect charge state that is not stable in a 0 K formation-energy diagram can acquire a stability region at finite temperature, because the relevant quantity is a free energy rather than only an energy minimum.

Another important aspect of defect modeling is comparison with experiment. Several defect types can have similar stability or lead to similar experimental signatures, so experimental signals do not always map uniquely onto one relaxed defect structure. Positron annihilation spectroscopy is a useful example.^89^ It is highly sensitive to vacancy-type defects, but microscopic assignments require calculated positron lifetimes and trapping characteristics.^90,91^ In lead halide perovskites, positron annihilation lifetime spectroscopy combined with density-functional theory enabled identification of lead vacancies in MAPbI_3_.^92^ Related combinations of positron annihilation spectroscopy, impedance spectroscopy, and first-principles calculations have been used to identify and suppress point defects in bromide perovskite detector materials.^93^

Defects can also directly affect surface reaction mechanisms. In BiVO_4_, surface oxygen vacancies can influence the oxygen evolution reaction. The neutral vacancy is thermodynamically unstable at the surface and spontaneously ionizes, while the ionized oxygen vacancy can reduce the thermodynamic overpotential for water oxidation.^69^ In Nb-doped TiO_2_(110), machine-learning-accelerated sampling of defect-polaron configurations showed that oxygen vacancies stabilize surface polarons that strongly affect CO adsorption, while Nb dopants mainly provide excess electrons.^94^

## 7 Interfaces

Semiconductor devices and photocatalytic systems contain interfaces, and this is where ideal-crystal models become least well-defined. At the same time, interfaces are where the relevant reaction occurs or where degradation may be initiated. Modeling an interface requires making choices about how the two materials meet, including their orientation, termination, and reconstructions. Several models may be chemically plausible and computationally stable, but correspond to different experimental situations. The first difficulty is therefore to construct a plausible and relevant interface model.

For applications in photocatalysis and photoelectrochemistry, semiconductor–water interfaces need to be represented explicitly because this is where charge transfer and surface reactions take place. Hybrid-functional calculations on TiO_2_ and BiVO_4_ have shown how localized holes at semiconductor–water interfaces affect the energetics of water oxidation.^68,95,96^ The relevant interface structure can also depend on the aqueous environment. For BiVO_4_(010), first-principles simulations showed that the surface coverage changes with pH. Molecularly adsorbed water dominates over a wide pH range, while hydroxyl adsorption becomes favorable under alkaline conditions.^97^ This pH-dependent surface chemistry can influence the preferred water-oxidation pathway when combined with the band alignment at the BiVO_4_–water interface.^98^ Many first-principles studies of semiconductor–water interfaces still rely on relatively simple, unreconstructed surface models. Recent machine-learning-accelerated surface exploration showed that BiVO_4_(010) can exhibit many reconstructed surface structures, suggesting that realistic simulations of surface reactivity may require broader sampling of surface configurations.^99^

Solid–solid interfaces introduce a different set of issues. In perovskite solar cells, the absorber is contacted by transport layers and modified by molecular passivation agents or self-assembled monolayers.^100–102^ From the computational perspective, it is useful to be able to assess quantities such as interface stability, band alignment, charge transfer, or adsorption energies. However, these quantities can depend strongly on how the interface model is constructed. One must decide how the two materials are matched, which surface termination is exposed, and whether defects or interfacial species are present.^103^ Even the bare surface of one of the contacted materials can be difficult to define. For MAPbI_3_, first-principles calculations showed that surface stability and electronic structure depend on whether the surface is flat or contains vacancies, and whether it is MAI- or PbI_2_-terminated.^104^ Thus, even before a contact layer is added, the electronic properties of the interface depend on which perovskite surface is assumed. A similar ambiguity appears for grain boundaries, whose electronic properties are determined by their precise local structure and composition. In halide perovskites, ideal grain-boundary models can appear electronically benign, whereas defect-rich or chemically modified boundaries can promote recombination, alter ion migration, or affect local band bending.^105–107^

## 8 Conclusions

Atomistic modeling is essential for identifying the mechanisms that control the properties of complex semiconductors. The examples discussed in this Perspective show how predictive modeling often requires going beyond a single ideal crystal structure. Depending on the property of interest, relevant effects can include local structural complexity, finite-temperature dynamics, compositional disorder, charge localization, defects, and interfaces.

A common theme is that these effects do not just add detail to an existing picture. In many cases, they change the interpretation of the calculation by modifying the relevant structure, charge state, reaction pathway, or measurable response. The challenge is therefore to identify which degrees of freedom need to be represented explicitly for a given question.

These examples can also be used as a practical guide when constructing an atomistic model or interpreting disagreement with experiment. The relevant question is whether the model represents the structural and electronic degrees of freedom that can affect the observable of interest. Depending on the problem, this may require considering local structural distortions, finite-temperature sampling, compositional disorder, charge localization, defects, or the interface environment. Convergence should then be assessed for the target property as well as for the underlying structural model.

Several of these effects can occur simultaneously. A photoelectrochemical interface, for example, can involve a reconstructed surface, liquid water, an applied potential, thermal motion, defects, and localized or excited carriers.^108^ Treating all of these effects at the same level in a single calculation is rarely feasible. In practice, different levels of theory and sampling can be combined, with the effects expected to control the target property treated explicitly and the remaining approximations tested separately. Quantitative estimates of accuracy, computational cost, and accessible time and length scales cannot be assigned universally. They depend on the material, target observable, system size, and convergence requirements.

Machine-learned interatomic potentials are becoming increasingly useful in this context because they allow larger configurational spaces to be sampled than is practical with direct electronic-structure calculations alone. Their reliability depends on whether the training data cover the relevant structures, compositions, and thermodynamic conditions. Active or on-the-fly learning and uncertainty estimates can help identify extrapolation and configurations for which additional reference calculations are needed.^109^ Long-range electrostatics and charge transfer may also require models that go beyond local atomic environments.^110^

Combined with first-principles methods, they provide a route toward more realistic models of complex semiconductors and simulations that are more closely connected to experimental conditions.
